# Universal digital mental health interventions for children and youth: a scoping review

**DOI:** 10.3389/fdgth.2025.1665975

**Published:** 2025-11-17

**Authors:** Kaitlin Di Pierdomenico, Oana Bucsea, Haleh Hashemi, Arianna Leguia, Anne Lovegrove, Robert Cribbie, Rebecca Pillai Riddell

**Affiliations:** 1 Department of Psychology, York University, Toronto, ON, Canada; 2 Strong Minds Strong Kids Psychology Canada, Toronto, ON, Canada

**Keywords:** universal, Tier 1, digital mental health intervention (DMHI), child and youth, e-mental health, equity

## Abstract

**Introduction:**

Digital mental health interventions (DMHIs) are increasingly used to support child and youth mental health, yet the scope and characteristics of universal (Tier 1) DMHIs remain poorly defined.

**Methods:**

This scoping review synthesized peer- reviewed studies of universal DMHIs delivered to children and youth aged 0–18 years.

**Results:**

Use of online programs and hybrid delivery were prevalent. Many programs used an independent structure, while facilitation varied across self- led, facilitator-led, and both-led models. Outcomes were commonly assessed across multiple domains, including emotional, behavioural, social, and cognitive outcomes. When single domains were examined, these most often focused on emotional outcomes such as anxiety and depression. Interventions frequently employed therapeutic approaches such as cognitive behavioural therapy and psychoeducation, with content emphasizing emotion regulation, coping and problem-solving skills, and mental health literacy. Structural features varied in length and number of sessions, and many included scaffolded self-managed elements such as messaging or check-ins. Early childhood was underrepresented, with limited reporting of child outcomes for ages 0–4. Equity considerations were also limited, as many studies did not report race or ethnicity and sex and gender reporting was often binary or unspecified. Youth involvement in intervention design or consultation was uncommon.

**Discussion:**

This review provides an overview of the current landscape and identifies key gaps, including limited equity considerations, the underrepresentation of early childhood development, and minimal youth involvement. Clearer reporting of delivery structure and facilitation, stronger demographic reporting, and component-focused evaluation can support equitable, developmentally responsive scale-up.

**Systematic Review Registration:**

https://inplasy.com/inplasy-2024-9-0026/, INPLASY202490026.

## 1 Introduction

### 1.1 Children and youth mental health needs

Mental health conditions typically emerge early in life: two-thirds to three-quarters develop before the age of 24 (1), with a median onset at 18 years and a peak at 14.5 years (2). Early-onset conditions frequently persist into adulthood, emphasizing the importance of prevention and early intervention to equip children and youth with effective coping strategies and reduce the likelihood of long-term challenges (3). The global decline in youth mental health during and after the COVID-19 pandemic further highlights the urgent need for population-level supports (3). The pandemic also exposed critical limitations of traditional service systems and accelerated the adoption of digital approaches (4).

### 1.2 Digital mental health interventions (DMHIs)

Digital mental health interventions (DMHIs) use technologies such as apps, web-based platforms, and online programs to deliver prevention, treatment, and mental health support, depending on their intended purpose and design (5, 6). They include self-guided or facilitated programs, asynchronous and synchronous formats, blended models combining online and offline components, and tools such as serious games (digital games designed for therapeutic or educational purposes) and virtual reality. DMHIs are particularly appealing for young people, who are highly digitally connected (7, 8), and they can reduce barriers such as stigma, geography, and wait times (9, 10). However, these benefits are not equitably distributed: gaps in access to devices, reliable internet, and digital literacy mean that youth from low-income, rural, or historically marginalized communities remain underserved (9, 11). Without equity-focused design, DMHIs risk reinforcing rather than reducing disparities (12).

### 1.3 Equity and marginalization

In this review, digital marginalization refers to the social, economic, and structural conditions that constrain access to digital mental health resources and opportunities. These conditions are shaped by factors such as race, ethnicity, sex, gender, socioeconomic status (SES), geography, and culture (13). Children and youth who experience marginalization often face barriers to participation in digital interventions, including limited access to devices or reliable internet, lower levels of digital literacy, and programs that lack culturally responsive design and content (14, 15). Such inequities have significant implication for the reach and effectiveness of universal DMHIs. Greater systematic attention to equity is therefore needed to assess whether universal DMHIs are achieving population-level impact.

### 1.4 Tiers of service delivery and universal approaches

DMHIs can be situated within a tiered model of mental health service delivery (16):

- Tier 1 (Universal): Interventions offered to all children and youth, regardless of circumstances, with the goal of promoting health, strengthening resilience, and preventing the onset of mental health difficulties.
- Tier 2 (Selective): Targeted supports for children and youth with elevated vulnerability or showing early signs of distress.
- Tier 3 (Indicated/Clinical): Intensive, individualized interventions for young people with identifiable or diagnosable mental health conditions.

This review focuses on Tier 1 DMHIs. While universal approaches are designed to strengthen health and resilience, many studies nonetheless evaluate outcomes such as anxiety and depression symptoms. This reflects a conceptual tension between the promotion/prevention goals of universal interventions and the symptom-focused measures used in much of the existing evidence base.

### 1.5 Theoretical grounding

Universal DMHIs can be conceptually anchored in several complementary frameworks. Prevention science emphasizes early, population-wide strategies to reduce incidence of mental health challenges (17). Developmental psychopathology highlights how mental health trajectories unfold across stages of growth (18). Ecological systems theory situates child development within multiple interacting contexts (family, school, community, and culture) (19). From this perspective, universal DMHIs have the potential to reduce emerging symptoms while simultaneously strengthening protective factors across development. For example, appropriate targets for Tier 1 DMHI would be:

- Early childhood (0–5): Foundational emotion regulation and caregiver-child interactions (20).
- Middle childhood (6–12): Coping skills, problem-solving, and peer relationships (21, 22).
- Adolescence (13–18): Identify development, resilience, stress management, and mental health literacy (23).

### 1.6 Existing reviews and gaps

Prior reviews of school-based or digital mental health programs report mixed effects, with some interventions demonstrating modest benefits and others limited or no impact (24). However, most of the literature has focused on Tier 2 (selective) or Tier 3 (clinical) interventions for children and youth with elevated symptoms or diagnosed conditions (10, 25, 26).

Reviews that include universal interventions often analyze them alongside targeted or clinical programs, making it difficult to isolate Tier 1 effects. For example, Bergin et al. (27) synthesized preventive digital interventions but grouped universal and selective programs together, limiting conclusions about population-level delivery. Similarly, Liverpool et al. (28) reviewed digital health interventions broadly, without distinguishing universal from clinical tiers, and equity considerations were minimally addressed. More recently, Piers et al. (13) focused on socioeconomically and digitally marginalized youth, highlighting critical access gaps, but did not provide a systematic overview of the broader universal DMHI landscape.

Consequently, the scope, content, and delivery of universal (Tier 1) DMHIs remain poorly defined. Key questions about developmental coverage, outcome domains, and equity, including representation of younger children and marginalized populations, have not been systematically addressed. This scoping review extends prior work by (1) focusing exclusively on universal interventions, (2) situating them within developmental and theoretical frameworks, and (3) foregrounding equity and inclusion as central dimensions of the evidence base.

### 1.7 Aim of this review

This scoping review addresses limitations in prior work by systematically characterizing the peer-reviewed literature on universal (Tier 1) DMHIs for children and youth aged 0–18. The review examines five key domains:

1. Populations studied: Which developmental stages and demographic groups are represented?
2. Objectives and outcomes: What outcomes are targeted and how are they measured?
3. Intervention parameters: What are the key design and implementation features?
4. Content and approaches: Which therapeutic and conceptual foundations are used?
5. Delivery models: How are interventions facilitated and by whom?

By synthesizing evidence across these domains, this review clarifies the current landscape of universal DMHIs, identifies gaps in developmental and equity coverage, and provides a foundation for the design, implementation, and evaluation of future Tier 1 programs.

## 2 Methods

**Figure 1:**
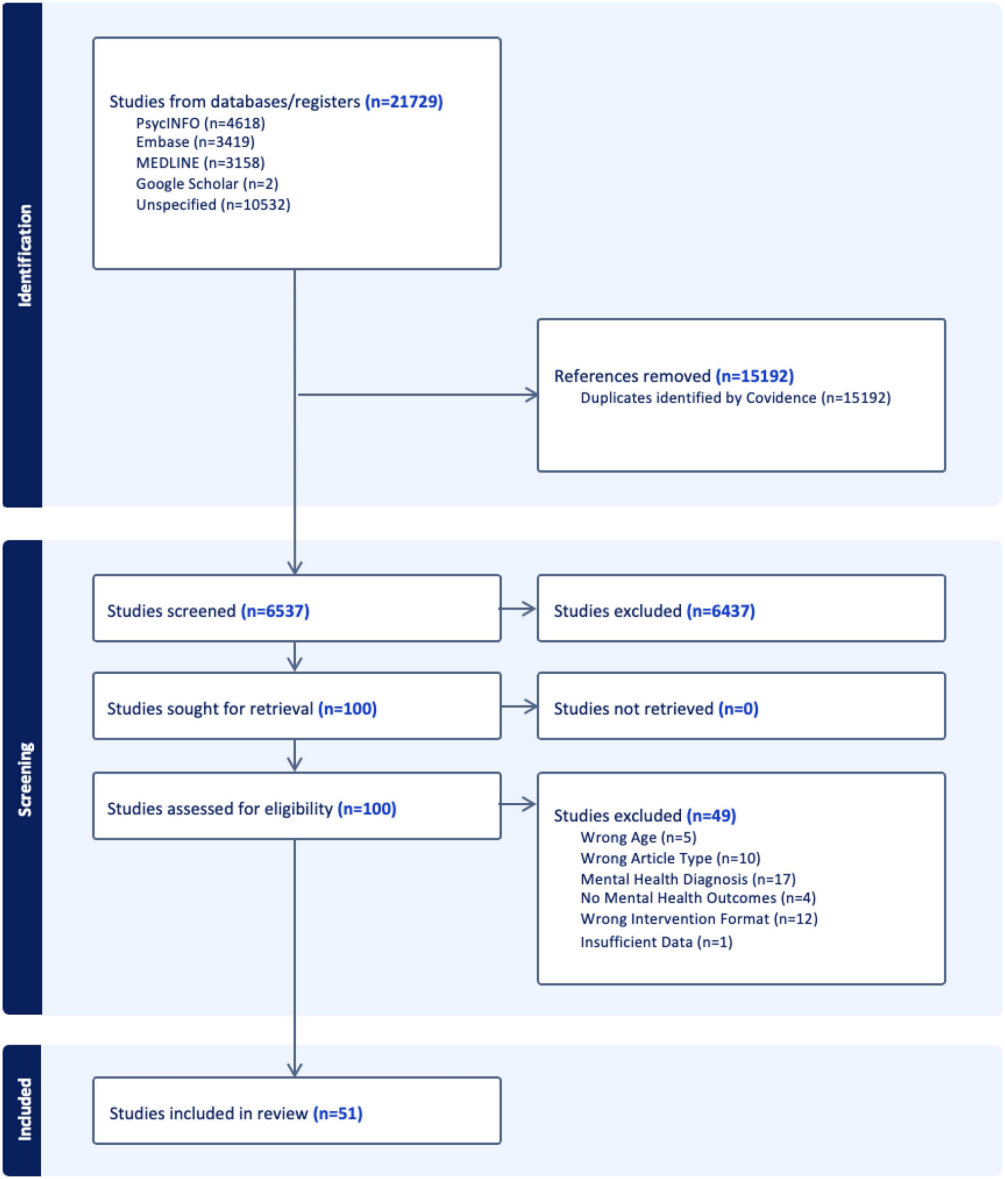
PRISMA flowchart.

This review was prospectively registered on the International Platform of Registered Systematic Review and Meta-analysis Protocols (**INPLASY Protocol 6765**). The conduct and reporting of the review followed the Preferred Reporting Items for Systematic Review and Meta-Analyses extension for Scoping Review (PRISMA-ScR) guidelines (29). The completed PRISMA-ScR checklist is available in the Supplementary Materials, and a PRISMA flowchart summarizing the study selection is provided in Figure 1.

### 2.1 eligibility criteria

#### 2.1.1 Types of sources

This review included full-text, peer-reviewed original research articles, as these represent the highest standard for evidence-based practice in health and social care. Non-peer-reviewed sources (e.g., book chapters, case studies, conference abstracts, and dissertations) were excluded.

#### 2.1.2 Participants

Studies were eligible if the minimum or mean age of participants was between 0 and 18 years. Studies that included participants over 18 were still considered if the average age of the sample was approximately 18 years or younger. Studies were excluded if they focused exclusively on adults or did not specify participant age.

#### 2.1.3 Mental health Status

Only studies involving non-clinical populations were included. Studies were excluded if they focused on participants who had been hospitalized for acute medical or psychiatric conditions (e.g., suicide attempt or cancer treatment) or had recently been discharged from such care.

#### 2.1.4 Intervention format

Studies had to include a digital intervention. Eligible interventions were either virtual (delivered entirely online, without in-person components) or hybrid (integrating digital tools with in-person elements, such as school-based implementation). Studies that relied solely on in-person interventions without a digital component were excluded.

#### 2.1.5 Measured outcomes

Studies were included if they measured at least one mental health outcome within the domains of emotional, behavioural, social, or cognitive. Studies that assessed only physiological measures without a corresponding mental health outcome were excluded. In this review, psychological outcomes were defined broadly to include:

- Emotional (e.g., mood, affect, anxiety, depression)
- Behavioural (e.g., conduct, coping actions)
- Social (e.g., peer relationships, connectedness)
- Cognitive (e.g., attention, executive function, problem-solving)

For each study, one outcome measure per domain (emotional, behavioural, social, cognitive) was selected for synthesis to support parsimony and future effect size synthesis. The decision was made to reduce heterogeneity between trials being synthesized, increase confidence in future syntheses, and prepare the dataset for subsequent meta-analysis. When more than one outcome per domain was reported, authors prioritized the most psychometrically valid measure. If multiple high-quality outcomes were reported, frequency of use amongst other included studies was examined. This approach also reduced redundancy, as studies that included several measures of the same construct (e.g., multiple anxiety scales) were represented only once per domain, preventing overweighting of particular outcomes or studies in the synthesis. Importantly, for each intervention, all outcome domains that were assessed were documented, even though only one measure per domain was carried forward into the synthesis.

#### 2.1.6 Search strategy and data sources

A systematic search for DMHIs targeting psychological outcomes was created with a highly experienced academic librarian. The search process began with a preliminary scan of Google Scholar to identify key articles relevant to the three main components of the research question: universal scope, DMHIs, and children and youth. These initial articles informed the development of a comprehensive list of keywords, which were refined to ensure compatibility across databases.

The final search strategy was implemented in three major databases: MEDLINE, PsycInfo, and Embase. An example of the search terms used across these databases is provided in Supplementary Table S2. The initial search was conducted between June 26, 2024, and July 5, 2024. To capture newly published literature, an updated search using the same strategy was performed on January 6, 2025, covering studies published between July 3, 2024, and January 6, 2025.

#### 2.1.7 Study selection

All search results were imported into Covidence, a reference management and screening tool, to facilitate de-duplication, title and abstract screening, full-text review, and data extraction. While many records were automatically categorized as database sources based on indexed journal information, a large proportion (*n* = 10,532) were labeled as “unspecified sources” due to missing metadata. This included manually uploaded references and records lacking identifiable source information.

Title and abstract screening was conducted by a four-person review team, with all records independently screened by two reviewers. Reliability screening was performed on a subset of studies (25% of the total sample) to assess consistency in screening decisions. Full-text articles deemed potentially relevant were uploaded to Covidence for further screening by two independent reviewers. Discrepancies in inclusion or exclusion decisions were resolved through weekly consensus meetings among the authorship team. Full-text data extraction was conducted using both Covidence and Excel, and always performed by two independent reviewers.

We also manually searched the reference lists of relevant excluded systematic reviews and conducted targeted handsearching of key journals and other sources likely to contain relevant studies. Inter-rater agreement for title and abstract screening ranged from 0.84 to 0.98, while agreement for full-text review ranged from 0.90 to 0.96, indicating an almost perfect agreement between reviewers (30).

#### 2.1.8 Data extraction

Data extraction was conducted using Microsoft Excel, with categories initially adapted from the PICO framework: Population, Intervention, Comparators, and Outcomes (31). Variables selected for extraction (as reported above) were part of an iterative process as familiarity with the scope of literature deepened. Data extraction templates were piloted and revised to improve clarity and consistency between co-authors. Each article was independently extracted by two reviewers to improve accuracy and reliability.

#### 2.1.9 Data synthesis

Descriptive statistics (e.g., frequencies, medians, and ranges) were used to summarize study characteristics. Data were presented in both tabular and graphical formats. Key variables synthesized included publication year, country of origin, study design, population demographics (e.g., age, marginalized status), DMHI characteristics (e.g., format, setting, facilitation), and mental health outcomes (e.g., domains, tools, reporting methods).

## 3 Results

### 3.1 Study selection and characteristics

A total of 21,729 studies were identified through the search strategy. After de-duplication and screening, 51 peer-reviewed studies on universal DMHIs for children and youth were included in the scoping review (Figure 1).

Across the 51 included articles, we identified 45 unique interventions (88%). Several programs were evaluated in more than one study and were therefore grouped together as intervention “families.” Specifically, SPARX/SPARX-R (32, 33), MoodGYM (34, 35), Hero (36, 37), Life-Fit-Learning/RISE (38, 39), Bite Back (40, 41), and OKmind (42, 43) were each evaluated in two separate articles. All other interventions were unique to a single article. Most studies were conducted in middle- to high-income countries (*n* = 49, 96%), primarily in North America (*n* = 12, 24%) and Australia (*n* = 12, 24%). Only two studies were conducted in low-income countries (44, 45). See Supplementary Table S1 for a summary of Study Characteristics of Universal DMHIs For Children and Youth.

All included studies were published since 2002, with the highest number published in 2024 (*n* = 10, 20%). The majority were published between 2021 and 2024 (*n* = 27, 53%). Study sample sizes ranged from 24 to 1,767 participants.

Most studies employed between-group designs while a notable minority used within group (i.e., repeated measures) designs to track changes within participants over time (*n* = 12, 24%). Randomized controlled trials (RCTs) were the most common study design (*n* = 35, 69%), followed by non-randomized interventional studies (*n* = 17, 33%). Many studies also included follow-up assessments after the initial intervention period to examine long-term effects (*n* = 23, 45%), but the number and duration of follow-ups varied notably. Follow-up time periods included: immediate (within 1 month post-intervention, *n* = 2), 1–3 months post-intervention (*n* = 14), 4–6 months post-intervention (*n* = 7), and 12-months or beyond (*n* = 5).

Study quality was assessed for all included studies using appropriate tools based on study design. The Cochrane Risk of Bias 2 (RoB 2) (46) tool was applied to RCTs, and the ROBINS-I tool (47) was used for non-RCTs. Among the 35 RCTs (69% of included studies), risk of bias was variable: 8 studies (22.9%) were rated as having low overall risk; 11 studies (31.4%) had some concerns; 16 studies (45.7%) had a high overall risk of bias. Common sources of bias included lack of participant and personnel blinding (performance bias), inadequate blinding of outcome assessors (detection bias), and issues related to attrition. Among the 16 non-RCTs (31%), most were pre-post single-group designs, with a smaller number using quasi-experimental comparisons. The ROBINS-I assessments indicated that: 14 studies (87.5%) had a serious overall risk of bias; 2 studies (12.5%) had a moderate risk. The most frequent concerns involved confounding, participant selection, and measurement of outcomes. Overall, the findings reflect a high degree of methodological variability and a substantial proportion of studies with notable sources of bias, particularly among non-randomized designs. This pattern reduces confidence in individual study findings and limits the certainty of conclusions that can be drawn from the current evidence base.

### 3.2 Results according to Key domains and research questions

1. **Populations studied:** which developmental stages and demographic groups are represented?

The features of target populations are summarized in Table 1. Of the 51 included studies, 45 (90%) targeted school-aged children and adolescents (5–18 years), while only four studies (8%) addressed preschool-aged children (0–4 years). These groupings were not predetermined based on developmental theory (e.g., 0–5, 6–12, 13–18) but instead reflected how populations were reported within the studies themselves. One study (2%) focused on children aged 4–7 years, which did not align neatly with either grouping (43).

**Table 1:** Key features of target populations.

Population features	No. of studies (%)	
Age		
5–18	46 (90)	
0–4	4 (8)	
Mixed (4–7)	1 (2)	
Participant Focus		
Child-Youth Involved Only	39 (76)	
Caregiver-Mediated	9 (18)	
All Female		5 (56)
Predominantly Female		3 (33)
All Male		1 (11)
Caregiver-Involved	3 (6)	
Unclear/Not-Reported		2 (67)
Predominantly Female		1 (33)
Sex		
Sex Balance		
Predominantly Female	16 (31)	
Balanced	15 (29)	
Predominantly Male	9 (18)	
Unclear/Not-Reported	6 (12)	
Exclusively Female	3 (6)	
Exclusively Male	2 (4)	
Sexual Orientation Reporting		
Not-Reported	48 (94)	
Reported	3 (6)	
Gender		
Gender Identity Reporting		
Unclear/Not-Reported	28 (55)	
Binary	19 (37)	
Acknowledged Non-Binary Categories	4 (8)	
Proportion of Racialized Participants		
No racial data provided	32 (63)	
0%–24%	10 (19)	
75%–100%	6 (12)	
25%–49%	3 (6)	
Most Prevalent Racialized Group	14 (27)	
Latin American		7 (50)
Indigenous		3 (22)
Black		2 (14)
East Asian		2 (14)
Lived Experience with Marginalization		
Non-Marginalized Communities	45 (88)	
Historically Marginalized Communities	6 (12)	

Across the included studies, 39 (76%) involved children and youth directly as participants, while a smaller proportion incorporated their caregivers, either through caregiver-mediated or caregiver-involved interventions. Caregiver-mediated interventions were delivered directly to parents or caregivers with the intention of influencing child outcomes (42, 45, 48–54). All interventions targeting children aged 0–4 years fell into this category (42, 45, 49, 50). Within the subset of caregiver-mediated interventions (*n* = 9, 18%), caregiver biological sex was reported as exclusively female in 5 studies (56%), predominantly female in 3 (33%), and exclusively male in 1 (11%). Caregiver-involved interventions (*n* = 3, 6%) included structured parent-child interaction components alongside child-focused programming (55–57). In these studies, caregiver biological sex was most often unclear or not reported (*n* = 2, 67%), with one study (33%) identifying caregivers as predominantly female (57).

Reporting on the biological sex of child and youth participants was also extracted. Sixteen studies (31%) reported predominantly female participants, 15 (29%) reported a balanced sex distribution, and 9 (18%) reported predominantly male participants. Six studies (12%) did not report or were unclear, 3 (6%) included only female participants, and 2 (4%) included only male participants. In terms of gender identity: 19 studies (37%) used binary categories (male/female), often conflating gender identity with biological sex; 4 (8%) acknowledged gender diversity beyond the binary (e.g., non-binary or transgender identities); and 28 (55%) did not specify how gender was defined or did not report it. Sexual orientation was rarely reported among caregivers or children/youth: 3 studies (6%) reported it, whereas 48 (94%) did not report.

To assess diversity within study samples, participants were also categorized according to the proportion of racialized individuals reported from a local perspective (i.e., populations considered racialized within the country where the study was conducted). Racial and cultural demographics were inconsistently reported overall: 32 studies (63%) provided no racial data. Among the 19 studies (37%) that did, most reported samples with 0%–24% racialized participants (*n* = 10, 19%), followed by 75%–100% (*n* = 6, 12%), and 25%–49% (*n* = 3, 6%). Of these 19, only 14 specified the most prevalent racialized group. Within this subset, the most commonly reported populations were Latin American (*n* = 7, 50%), Indigenous (*n* = 3, 22%), Black (*n* = 2, 14%), and East Asian (*n* = 2, 14%), together representing 100% of the 14 studies that reported such details.

Representation from historically marginalized communities was further assessed based on whether studies explicitly targeted these populations (e.g., interventions designed for participants with low SES status or specific racial or cultural identities). Most studies did not explicitly focus on marginalized populations (*n* = 45, 88%). A smaller proportion (*n* = 6, 12%) reported including participants who experience systemic barriers. Within this subset, areas of marginalization included racialized and ethnically diverse youth (*n* = 3), low-income populations (*n* = 3), caregivers in low-resource settings (*n* = 2), and Indigenous communities (*n* = 1). Several interventions addressed overlapping forms of disadvantage, such as race and economic status. For example, studies included Latinx sexual minority youth (56), Black and biracial adolescent girls from low-income urban schools (58), and low-income Latino and African American adolescents (59). Other studies targeted economically disadvantaged adolescents more broadly (60), caregivers of young children in Zambia and Tanzania (45), and Inuit youth in remote Nunavut communities (32).

1. **Objectives and outcomes**: what outcomes are targeted and how are they measured?

Table 2 summarizes the target objectives and outcomes of the included studies. Nineteen studies (37%) were primarily prevention-focused, 18 (35%) were promotion-focused, and 14 (28%) addressed both aims. Prevention interventions were defined as those designed to reduce the onset or escalation of symptoms (e.g., teaching thought challenging to decrease worry). Promotion interventions were defined as those aiming to enhance positive functioning (e.g., improving social skills or help-seeking). Some interventions explicitly combined promotion and prevention aims, such as programs that taught skills for making friends while also including distortion identification strategies to prevent fear of negative evaluation (61).

**Table 2:** Key features of target objectives and outcomes.

Outcome features	No. of studies (%)
Objective	
Prevention	19 (37)
Promotion	18 (35)
Both (Promotion & Prevention)	14 (28)
Outcomes	
Multiple	29 (57)
Emotional	14 (27)
Behavioural	4 (8)
Social	3 (6)
Cognitive	1 (2)
Reporting	
Self-report	35 (69)
Caregiver-report or teacher-report	9 (17)
Combination of self- and caregiver-/school- reports	7 (14)

Interventions targeted a range of psychological outcomes, including emotional (e.g., how participants feel), behavioural (e.g., how participants act), social (e.g., how participants connect with others), and cognitive (e.g., how participants think or solve problems). Most studies assessed multiple outcome domains (*n* = 29, 57%). Fourteen studies (27%) focused solely on emotional outcomes, four (8%) on behavioural outcomes, three (6%) on social outcomes, and one (2%) exclusively on cognitive outcomes (44).

All but two studies employed validated measures, defined as standardized tools that have undergone psychometric testing for reliability and validity, such as the *Revised Children's Manifest Anxiety Scale* (RCMAS) (62) or the *Child Behaviour Checklist* (CBCL) (63). Two studies also included additional unvalidated, study-specific instruments, developed for the intervention and not independently validated. Examples include Chillemi et al.'s (64) questionnaire on coping strategies for cyberbullying and Lang et al.'s (59) *Computeen Computer Skills Inventory* for assessing digital skills.

Self-report was the most common reporting method (*n* = 35, 69%), where participants provided their own feedback after the intervention. Caregiver or teacher report was less common (*n* = 9, 17%), though notably, all four interventions delivered to children aged 0–4 relied exclusively on caregiver reports. A smaller number of studies used a combination of self- and caregiver/teacher reporting (*n* = 7, 14%).

1. **Intervention parameters**: what are the key design and implementation features?

The characteristics of intervention parameters, including format, design, and structure, are summarized in Table 3. The majority of studies utilized hybrid formats (*n* = 27, 53%), where a digital intervention was supplemented by an in-person component (e.g., students accessing an online program with teacher facilitation at school). This was followed by fully virtual interventions (*n* = 20, 39%) and a small number of studies that included both hybrid and virtual intervention arms (*n* = 4, 8%). Among hybrid DMHIs, the in-person component most frequently took place in school settings (*n* = 28, 90%).

**Table 3:** Key features of intervention parameters.

Parameter features	No. of studies (%)	
Format		
Hybrid	27 (53)	
Virtual	20 (39)	
Mixed (Hybrid + Virtual Arms)	4 (8)	
Design		
Online Program	24 (47)	
App	9 (17)	
Virtual Communication	6 (12)	
Virtual Communication + Website	6 (12)	
Video Game	3 (6)	
Website	2 (4)	
Virtual Reality	1 (2)	
Structure		
Independent Work	23 (45)	
Group-Based & Independent Work	15 (29)	
Group-Based & Independent Work & One-to-One	6 (12)	
Independent Work & One-to-One	5 (10)	
Group-Based	2 (4)	
Youth Involvement in Intervention Design		
Youth Involvement	8 (16)	
Youth-Consulted/User-Informed		5 (10)
Youth Co-Design		3 (6)

Most interventions were online programs (*n* = 24, 47%), typically delivered through platforms or portals. This was followed by apps (*n* = 9, 17%), standalone software applications designed for mobile devices or computers (e.g., meditation apps). Some interventions used virtual communication tools (*n* = 6, 12%), such as video conferencing or chat platforms for real-time interaction, while others combined virtual communication with websites (*n* = 6, 12%) to provide additional resources, modules, or educational content. Fewer interventions relied solely on websites (*n* = 2, 4%) or incorporated video game elements (*n* = 3, 6%). Only one study implemented virtual reality as the primary intervention format (65).

Intervention structure varied across studies. Structure refers to the format through which participants engaged with intervention content, independent of who facilitated delivery. Nearly half of the studies (*n* = 23, 45%) used independent work, where participants completed modules or activities on their own, sometimes supplemented with asynchronous check-ins. Fifteen studies (29%) used a combined format of independent work plus group-based sessions, allowing participants to complete self-directed activities alongside scheduled group discussions. Six studies (12%) implemented a multi-modal structure, integrating independent, group-based, and one-to-one sessions. In five studies (10%), the structure was independent work plus one-to-one sessions, where participants engaged in self-directed tasks and also attended individualized sessions with a facilitator. The least common structure was group-based only (*n* = 2, 4%), consisting of structured discussions or activities without a self-directed component.

In contrast to structure, facilitation was also examined. Facilitation (self-led, facilitator-led, or both-led) indicates who provided guidance or oversight. For example, an intervention may have an independent structure yet be “both-led” if participants complete modules independently after receiving facilitator instruction or check-ins.

Eight studies (16%) reported involving youth in the design of interventions. In five studies (10%), young people were consulted or informed through activities such as surveys, focus groups, or usability testing, where their input contributed to refining the interventions. Three studies (6%) reported youth co-design, in which young people partnered more directly in creating elements of the content, structure, or delivery features. Overall, the small number of studies involving youth highlights a substantial gap in participatory design within universal DMHIs.

1. **Content and approaches**: what therapeutic and conceptual foundations are used?

The characteristics of the interventions varied across studies, as summarized in Table 4. Regarding the number of sessions, most interventions without scaffolding included 6–9 sessions (*n* = 15, 29%) or 10 or more sessions (*n* = 12, 23%). Shorter interventions with 1–5 sessions and no scaffolding appeared less frequently (*n* = 8, 16%). Several studies used a *scaffolded self-managed* format, which involved a defined number of structured sessions or modules, along with supplementary support elements. These supports often included asynchronous check-ins, WhatsApp or SMS messages, discussion groups, or app-based nudges designed to maintain engagement, answer questions, or reinforce learning. For example, six studies (12%) included scaffolded self-management alongside 6–10 structured sessions, three studies (6%) combined 2–5 sessions with ongoing support, and two studies (4%) paired a single core session with additional communication. In contrast, fully self-managed programs (*n* = 5, 10%) allowed participants to access the intervention entirely on their own, at their preferred time and pace, without any additional support.

**Table 4:** Key features of intervention examined.

Intervention features	No. of studies (%)
Number of Sessions	
6–9 No Scaffolding	15 (29)
10+ No Scaffolding	12 (23)
1–5 No Scaffolding	8 (16)
Scaffolded Self-Managed + 6–10 sessions	6 (12)
Fully Self-Managed	5 (10)
Scaffolded Self-Managed + 2–5 sessions	3 (6)
Scaffolded Self-Managed + 1 session	2 (4)
Duration of Intervention in Hours	
Scaffolded Self-Managed (7–14 h)	10 (20)
7–14 No Scaffolding	10 (20)
1–6 No Scaffolding	9 (18)
Fully Self-Managed	9 (18)
15+ No Scaffolding	6 (12)
Scaffolded Self-Managed (1–6 h)	5 (10)
0–1 No Scaffolding	2 (4)
Therapeutic Approach	
Cognitive Behavioural Therapies	27 (53)
Psychoeducation	14 (27)
Other (Peer Support & Storytelling)	4 (8)
Behavioural Therapies	4 (8)
Mindfulness	2 (4)
Intervention Content *studies featured a range of intervention content	
Emotion Regulation	44 (86)
Coping & Problem Solving Skills	38 (75)
Mental Health Literacy	23 (45)
Social Skills	12 (24)
Parenting Skills	6 (12)
Attention/Focus/Executive Functioning	2 (4)

* Each study may have employed multiple types of content.

Program duration also varied. Many interventions were estimated to last between 7 and 14 h, whether through structured content delivery (*n* = 10, 20%) or scaffolded self-managed formats (*n* = 10, 20%), where added features such as messaging or peer chat contributed to total engagement time. Nine studies (18%) reported durations of 1–6 h, including scaffolded formats (*n* = 5, 10%), while fully self-managed programs also commonly fell within this range (*n* = 9, 18%). Fewer programs were longer (15 + h; *n* = 6, 12%) or very brief (under 1 h; *n* = 2, 4%) without scaffolding.

Cognitive Behavioural Therapies (CBT) were the most widely used therapeutic approach (*n* = 27, 53%), typically combining cognitive strategies (e.g., thought restructuring) with behavioural techniques (e.g., goal-setting, exposure). Psychoeducational approaches (*n* = 14, 27%) focused on increasing awareness of mental health and self-care strategies. A smaller number of studies used behavioural therapies alone (*n* = 4, 8%), while others employed mindfulness-based approaches (*n* = 2, 4%) or peer-support and storytelling methods (*n* = 4, 8%), including digital storytelling and peer-led discussions.

Intervention content addressed a broad range of skill areas. Emotion regulation was the most frequently targeted area (*n* = 44, 86%), with strategies designed to help participants understand, express, and manage emotions. Coping and problem-solving skills (*n* = 38, 75%) were also prominent, supporting youth in handling stress and navigating challenges. Mental health literacy was addressed in nearly half of the studies (*n* = 23, 45%), aiming to build understanding, reduce stigma, and encourage help-seeking. Fewer interventions emphasized social skills (*n* = 12, 24%), with content focused on promoting prosocial behaviours. Parenting skills (*n* = 6, 12%) were also included in some programs to support caregivers in fostering positive relationships and responsive caregiving. Only two interventions (*n* = 2, 4%) explicitly targeted attention, focus, or executive functioning through attention regulation practices and task initiation strategies.

1. **Delivery models**: how are interventions facilitated and by whom?

Table 5 summarizes the key features of who facilitated the intervention. Interventions were categorized as self-led, facilitator-led, or both-led. Self-led interventions allowed participants to independently access on-demand content, such as mobile apps or video games. Facilitator-led interventions involved real-time guidance from a trained mental health professional, such as in virtual therapy sessions or structured group programs. Both-led interventions included an initial facilitator-guided phase before transitioning to independent engagement. The majority of interventions were both-led (*n* = 35, 69%), while fewer were fully self-led (*n* = 12, 23%) or solely facilitator-led (*n* = 4, 8%).

**Table 5:** Key features of intervention delivery.

Delivery features	No. of studies (%)
Delivery	
Both-led (self- and facilitator-led)	35 (69)
Self-led	12 (23)
Facilitator-led	4 (8)
Facilitator Type	
Research Personnel	11 (29)
Registered Mental Health Professional	7 (18)
Teachers	6 (16)
Registered Mental Health Professionals & Child & Youth Workers & Counselors	5 (13)
Non-Registered Mental Health Professional	4 (11)
Not Described	2 (5)
Child & Youth Workers & Counselors	2 (5)
Mixed	1 (3)
Facilitator Involvement	
Teaching/Counseling	26 (67)
Non-Involved Resource	13 (33)

Of the 51 studies included in this review, 39 (76%) involved a facilitator in some fashion. Research personnel were the most frequently involved facilitators (*n* = 11, 29%), consisting of researchers or academic staff without clinical qualifications. Registered mental health professionals (*n* = 7, 18%), including licensed psychologists, psychiatrists, and clinical social workers, were also commonly involved. Teachers (*n* = 6, 16%) played a significant role in several studies, often integrating mental health interventions into their school curriculum. Some studies used multidisciplinary teams (*n* = 5, 13%), where licensed mental health professionals worked alongside child and youth workers or counselors. Other studies relied on non-registered mental health professionals (*n* = 4, 11%), such as peer supporters, wellness coaches, or unlicensed counselors. A small number of studies (*n* = 2, 5%) did not specify who facilitated the intervention, while another small number of studies (*n* = 2, 5%) were exclusively facilitated by child and youth workers and counselors. In addition, one study involved a mix of teachers and non-registered mental health professionals (66).

The level of facilitator involvement varied across studies. In most cases, facilitators were engaged in teaching or counseling (*n* = 26, 67%). This included providing direct instruction, delivering therapeutic guidance, or offering structured support through both group and one-on-one sessions. Their roles often involved leading psychoeducational sessions, conducting mental health counseling, and facilitating discussions and interactive activities. In contrast, some facilitators served as non-involved resources (*n* = 13, 33%). In these cases, facilitators did not actively guide the intervention but instead provided Supplementary Materials or background support. Their involvement included being available to answer questions, overseeing access to digital resources, or offering general guidance without directly interacting with participants.

## 4 Discussion

This scoping review describes the current state of the evidence base on universal (Tier 1) digital mental health interventions (DMHIs) for children and youth aged 0–18 years. While prior reviews have explored DMHIs for young people aged 10–24 years (67) or combined universal, selective/at-risk, and clinical samples (27), most existing scoping reviews focus either on adults (68) or on children and youth within clinical populations (69). By focusing exclusively on universal DMHIs for children and youth, this review identifies patterns in target populations, outcomes, intervention design/structure, and delivery, and highlights developmental and equity gaps that warrant attention, especially given the rapid expansion of DMHI research following the pandemic.

Across included studies, 90% focused on participants aged 5–18 years, with comparatively limited attention to early childhood (0–4 years). All 0–4 interventions were caregiver-mediated, with caregivers as the primary users and reporters of child outcomes. The gap identified here concerns the scarcity of evaluated universal DMHIs that report child outcomes for ages 0–4, rather than the absence of caregiver-facing tools. Several features constrain inference in this subset: outcomes were exclusively caregiver-reported (introducing reporter/expectancy bias), child measures were heterogeneous and not always developmentally sensitive to the 0–4 period, and studies rarely specified caregiver role parameters (training, intensity, fidelity), limiting reproducibility and scale estimates. Virtual platforms are not merely convenient alternatives but can serve as lifelines for caregivers who face barriers to attending in-person services (e.g., travel, scheduling, childcare). Although broader literature links early family supports and responsive caregiving to socioemotional development (42, 45, 49, 50) and highlights the plasticity and vulnerability of early development (70), the current Tier 1 evidence base for digitally delivered, caregiver-mediated programs with child outcomes remains thin and inconsistently described.

Most interventions paired a digital tool (e.g., app or online module) with some in-person support (e.g., teacher or facilitator activities), indicating that a hybrid delivery format was most studied. Interventions typically comprised six or more sessions (∼7–14 h) and often incorporated scaffolded self-management (e.g., asynchronous check-ins, prompts, SMS/WhatsApp reminders). Few studies isolated the effects of scaffolding, session intensity, or duration, so the optimal dose and format for universal programs remain unclear. To interpret and implement Tier 1 DMHIs, two orthogonal dimensions require clear reporting: structure and facilitation. This distinction explains why independent work was the most common structure (45%) even though self-led was less frequent as a facilitation mode (23%). For example, a hybrid program may have an independent structure (participants complete modules on their own) while facilitation is both-led (self-directed use paired with in-person sessions and support available during online use). Accordingly, future studies should clearly report both dimensions and, where possible, evaluate variations in facilitation and scaffolding to inform practical guidance on time-on-task and delivery formats for Tier 1 interventions.

Most studies assessed multiple psychological domains (emotional, behavioural, social, cognitive), whereas single-domain studies most often emphasized emotional symptoms (e.g., anxiety, depression). A further issue is measurement fit. Many studies used symptom-oriented clinical scales that are well validated for treatment samples but can be insensitive in universal populations (e.g., floor effects, low variance) and do not capture promotion targets (skills, functioning, literacy). Promotion outcomes are typically assessed with capability- or performance-focused tools (e.g., coping or emotion-regulation skills, social competence, executive function, mental health literacy), which differ conceptually and psychometrically from symptom scales. In the synthesis, all domains assessed were recorded; when multiple tools indexed the same domain within a study, one validated measure per domain per study was selected to enhance comparability and reduce redundancy, an approach that may nevertheless privilege symptom measures over asset-focused constructs. It is recommended to pair symptom scales with developmentally appropriate, population-referenced measures and to report simple distributional checks (e.g., proportion at minimum scores, variance).

Only 12% of studies explicitly targeted marginalized populations, and 63% did not report racial/ethnic demographics. Sex and gender reporting showed similar limitations: 37% used binary gender categories, 55% did not specify their approach or did not report gender, and only 8% acknowledged non-binary gender identities. Sex balance was typically reported for child/youth participants, but often within binary frames; sexual orientation was rarely reported (6%). These gaps extended to caregiver data in the few caregiver-focused studies. These gaps constrain inferences about who is reached and who benefits, and hinder cultural adaptation. To support equitable implementation at scale, the field needs standardized demographic reporting (race/ethnicity, gender identity, sex where appropriate, sexual orientation, socioeconomic indicators, geography). Programs must be culturally and linguistically adapted, include strategies to address digital divides (e.g., low-bandwidth modes, offline functionality, device access supports), and attend to gender identity and sexual orientation diversity. Few studies documented such adaptations, highlighting a missed opportunity to design DMHIs that are both inclusive and scalable. Attention to cultural barriers, such as mistrust of mental health systems, stigma, and lack of content in users' first languages, remains particularly underdeveloped.

Youth perspectives were rarely incorporated into intervention development, with only 16% of studies involving young people through consultation or co-design. This limited engagement raises concerns about whether interventions adequately reflect the realities, preferences, and digital practices of their intended users. Co-design with youth is critical, given the central role of trust and relevance in sustaining use of DMHIs (71, 72). Similarly, participatory and peer components were used infrequently. Here, peer refers to adolescents who share salient characteristics with the target users (e.g., age, school context, community) and who take structured roles within the intervention — for example, peer mentors, moderators, or coaches who facilitate discussions, model skills, or provide support. Expanding these participatory and peer-support elements represents an opportunity to improve acceptability, especially for groups historically underserved and underrepresented by traditional designs.

Interventions most commonly drew on CBT, followed by basic psychoeducation, with content frequently emphasizing emotion regulation, coping/problem-solving, and mental health literacy. Fewer interventions targeted social skills, parenting practices, or attention/executive functioning domains relevant to social learning and self-regulation. This matters in light of evidence that pandemic-related isolation disrupted early social-cognitive development, particularly in lower-SES groups (73), and that rising digital engagement may reduce opportunities to practice interpersonal skills (74). Despite widespread youth engagement with interactive media, video games (8%) and virtual reality (2%) were rarely used as standalone Tier 1 interventions. Testing these modalities, especially when combined with participatory and peer-based elements, may improve engagement and relevance.

Within a tiered service model, universal Tier 1 DMHIs are low-intensity, high-reach supports designed for non-clinical populations. By strengthening foundational skills across developmental stages, they can reduce emerging symptoms while promoting protective factors, and create low-friction pathways to selective and indicated supports (Tier 2/3) through education, screening, and referral prompts (16). Importantly, children and youth are not a homogeneous group, and not all will have the access, readiness, or interest needed to benefit from digital tools. DMHIs should therefore be seen as one component of a broader, layered system of promotion and prevention rather than a one-size-fits-all solution.

Although many studies employed randomized designs, methodological variability and persistent risk-of-bias concerns, particularly in non-randomized trials, temper confidence in individual findings. Clearer outcome specification, developmentally appropriate measurement, full demographic reporting, and rigorous implementation descriptions will improve interpretability and the practical utility of future universal DMHI evidence.

## 5 Conclusion

This scoping review synthesizes evidence on universal Tier 1 digital mental health interventions for children and youth aged 0–18. Hybrid models are common, but therapeutic approaches, delivery structures, and target outcomes remain heterogeneous. Three cross-cutting gaps warrant action. Developmental coverage is uneven, especially for ages 0–4 where caregiver-mediated delivery is appropriate but child-reported outcomes are rarely collected. Equity and inclusion are under addressed, with inconsistent demographic reporting, limited cultural or linguistic adaptation, and infrequent youth participation. Reporting and evaluation practices also constrain interpretability and planning for scale, including unclear separation of delivery structure and facilitation in many papers and limited testing of format, dose, scaffolding, or immersive and interactive modalities.

Next steps align with these gaps. Expand early-childhood work using developmentally sensitive child measures within caregiver-mediated models. Advance digital health equity by increasing representation, standardizing demographic reporting, and addressing access barriers. Compare hybrid and fully virtual formats to isolate the active components of in-person support. Broaden therapeutic approaches beyond CBT to include peer support, storytelling, and other youth-aligned modalities. Target promotion outcomes such as social skills and attention regulation alongside symptoms. Invest in the development and rigorous testing of immersive and interactive tools, for example gamified programs and virtual reality, to assess feasibility, engagement, and impact.

These findings also have practical implications. Educators can integrate independent-structure programs into classroom routines with brief facilitator touchpoints and provisions for digital inclusion. Clinicians and school mental health teams can position universal DMHIs as a first step in stepped care, linking brief digital engagement to simple screening and timely referral. Policymakers and funders can set minimum expectations for demographic and implementation reporting, support trials that pinpoint the delivery components most responsible for engagement and outcomes, and resource co-design and cultural and linguistic adaptation. Implemented in this way, universal DMHIs can be developmentally responsive, inclusive, and scalable within a layered system of promotion and prevention.

### 5.1 Limitations

This review has several limitations. We restricted inclusion to peer-reviewed publications, which excludes grey literature and ongoing or unpublished programs. Classifying interventions as “universal” was constrained by variable reporting of participant characteristics and scope. The age criterion (mean age 0–18) excluded transitional-age youth 18–25 who are often included in broader youth mental health frameworks. Comparability was further limited by heterogeneity in measures and outcomes, frequent reliance on caregiver-reported outcomes in 0–4 studies, incomplete demographic reporting, and inconsistent descriptions of implementation details, including facilitator training, delivery schedule, and intervention fidelity/adherence.

Despite these constraints, the review provides a systematic overview of how universal DMHIs are being conceptualized and implemented for children and youth. Closing gaps in early-childhood evidence and digital health equity remains essential for scalable and equitable uptake.

## Data Availability

The original contributions presented in the study are included in the article/Supplementary Material, further inquiries can be directed to the corresponding authors.
