# Supplementary material for: Universal digital mental health interventions for children and youth: a scoping review
Source: Front Digit Health. 2025 Nov 17;7:1665975. doi: 10.3389/fdgth.2025.1665975 (PMC12665752; doi:10.3389/fdgth.2025.1665975)
Supplement: Supplementary file 1 [file Datasheet1.pdf]

Supplemental Online Content

Supplementary Table 1. Study Characteristics of Universal DMHIs for Children and Youth

Ref.	Country	Total Sample	Comparators	Study Design	Study Quality	Hybrid Study Setting	Intervention Description	Study Focus	Reporting of Outcome	Measurement Scales of Outcomes Selected
Benzi, 2023	Italy	24	Repeated-measures design	Non-RCT	low	N/A	The eCONNECT Parent Group is a 10-session online attachment-based intervention aimed at strengthening parenting skills and improving parent-adolescent relationships. Delivered via video conferencing, it focuses on improving parental sensitivity, reflective functioning, and emotion regulation through guided discussions, role-plays, and reflective exercises.	The reduce attachment insecurity and improve parent-adolescent interactions.	Caregiver-report	Ontario Child Health Study Emotional Behavioural Scales
Bohr, 2023	Canada	48	Wait-list control	RCT	low	N/A	SPARX is a digital serious game designed to teach cognitive behavioural therapy (CBT) strategies to Inuit youth (ages 13-18) in Nunavut, Canada. The intervention was primarily self-led, with support from community facilitators. Through a fantasy-based environment, participants engaged in interactive challenges that helped them develop emotional regulation skills, resilience, and adaptive coping strategies.	To reduce hopelessness and depressive symptoms while improving resilience and cognitive coping strategies.	Self-report	Center for Epidemiological Studies Depression Scale Revised (CESD-R); The Highly Sensitive Child Scale (HSC)
Calear, 2009	Australia	1,477	Wait-list control	RCT	high	School	MoodGYM is a free, interactive, internet-based cognitive-behavioral therapy (CBT) program designed to improve mental health literacy and resilience. It consists of five sequential modules incorporating information, animated	The improve students' understanding of mental health concepts and develop.	Self-report	Revised Children's Manifest Anxiety Scale (RCMAS)

							demonstrations, quizzes, and exercises. Delivered over five weeks in a classroom setting, each module was presented weekly during a designated class period, with access restricted to prevent students from skipping ahead or altering responses.	coping strategies through structured online learning.		
Calear, 2016	Australia	1,767	Wait-list control and e-GAD health service condition (teacher-supported intervention)	three-arm cluster stratified RCT	low	School	The e-GAD school intervention is a school-based, online cognitive-behavioral therapy (CBT) program. Participants completed six self-guided online modules covering psychoeducation, cognitive restructuring, problem-solving, and relaxation techniques. The program included interactive exercises and quizzes to reinforce learning, and students were encouraged to practice skills between sessions.	To reduce generalized anxiety symptoms in adolescents.	Self-report	General Anxiety Disorder-7
Cheng, 2024	China	264	Wait-list control	RCT	high	N/A	The Digital Netizen Alliance (D.N.A.) program is a multicomponent positive psychology intervention. The program is structured around the P-A-G-E framework, which focuses on developing four key psychological skills: Psychological resilience, active coping, growth mindset, and emotion regulation. Delivered over four weeks, the intervention includes animated psychoeducational clips, mini-games for knowledge reinforcement, and gamified elements like badges and trophies. Students also participated in a collaborative pledge activity with their parents to encourage a balanced digital lifestyle. The	To prevent gaming disorder (GD) and enhance mental wellness among Chinese primary school students.	Self-report	Emotion Regulation Questionnaire for Children and Adolescents

							intervention was designed to be self-paced and accessible from home via digital devices, with facilitators available for support and progress monitoring.			
Chillemi, 2020	Australia	54	Repeated-measures design	Non-RCT	low	School	The Increasing Resilience to Cyberbullying (IRCB) program is a classroom-based online intervention aimed at improving adolescents' coping skills for cyberbullying. It focuses on three core strategies: self-compassion, cognitive behavioral therapy (CBT)-based reframing of negative thoughts, and recognizing the value of seeking professional mental health support. The program was designed to be completed individually within a single 60-minute classroom session and included psychoeducation, guiding examples, vignettes, and open-ended questions.	To increase students' ability to manage cyberbullying experiences by increasing self-kindness, reducing negative thought patterns, and promoting help-seeking behaviours.	Self-report	General Help-Seeking Questionnaire (GHSQ); Attitudes Toward Seeking Professional Psychological Help Scale (ATSPPH-S); Measure participants' use of strategies to cope with cyberbullying
DeGarmo, 2019	United States	426	Wait-list control	RCT	high	N/A	The Fathering Through Change (FTC) intervention is a self-led, online parenting program for divorced or separated fathers of children aged 4-12 years. The 10-module program covers communication, discipline, emotion regulation, problem-solving, and conflict resolution, using video lessons, interactive exercises, and instructional materials. Fathers received automated reminders to encourage participation.	To reduce coercive parenting, improve father-child relationships, and support child adjustment by improving parenting skills, emotional regulation, and conflict resolution strategies.	Caregiver-report	Strengths and Difficulties Questionnaire (SDQ); Prosocial Behaviour

DeSmet, 2018	Belgium	216	Control condition	RCT	high	School	The Friendly ATTAC game is a digital intervention designed to promote positive bystander behaviour in adolescent cyberbullying situations. Set within a narrative-driven gaming environment, players assume the role of a time-traveling character from the future who must navigate a school setting and make decisions on how to respond to cyberbullying on social media. Playing the game took less than 30 min to play.	To improve bystander intervention skills, increase confidence in responding to cyberbullying, and reduce passive or reinforcing behaviours in online bullying situations.	Self-report	KIDSCREEN
Estrada, 2024	United States	30	Control condition	RCT	low	University offices and later via web conferencing due to Covid-19	Familias con Orgullo (FcO) is a family-based intervention among Latinx sexual minority youth (SMY) aged 13-17 and their parents. In the 14-session program adolescents participated in group sessions focusing on coping with discrimination, emotion regulation, goal setting, and sexual health education. They also attended family sessions with their parents to work on communication, mental health, and conflict resolution. Parents attended group sessions to learn affirmative support strategies, parenting skills, and culturally relevant discussions on familismo and machismo. They also participated in family sessions to practice communication and strengthen family bonds.	To prevent drug use, sexual risk behaviours, and depressive symptoms among Latinx sexual minority youth (SMY).	Self-report	Center for Epidemiological Studies Depression Scale (CES-D)
Fridrici, 2007	Germany	267	Wait-list control	RCT	low	School	The intervention is a school-based stress management program delivered to 7th and 8th grade students. Psychologists led a core module introducing fundamental	To reduce stress levels.	Self-report	SSKJ3-8

							stress management concepts, with three optional modules focusing on cognitive strategies, social support, and relaxation/time management, allowing participants to tailor their learning. Additionally, an e-learning component (SNAKE) was available to some students, providing interactive exercises and summaries to reinforce session content.			
Gefter, 2024	United States	179	Remote; hybrid; and in-person conditions	Non-RCT	low	School	The Stanford Youth Diabetes Coaches Program (SYDCP) an 8-week school-based health promotion intervention aimed at adolescents (9th and 12th graders) from low-income communities. A "train the trainer" program in which health care professionals and trainees teach healthy high school students to coach family members with chronic health conditions, enhancing their health knowledge, psychosocial skills (self-esteem, self-efficacy, and problem-solving), and health behaviours like nutrition, physical activity, and stress management. It was delivered in remote and hybrid formats to accommodate different learning environments.	To promote health knowledge, psychosocial assets (self-esteem, self-efficacy and problem-solving) and health behaviours (physical activity, nutrition and stress reduction).	Self-report	Rosenberg Scale for Self-Esteem; CHKS; validated ability to manage stress questionnaire developed by authors
Goodman, 2014	United States	60	Repeated-measures design	Non-RCT (quasi-experimental study)	high	School	Ninth and twelfth-grade girls participated in either an oral storytelling group (OST), where they shared personal stories about stressors in a facilitated group setting, or a digital storytelling group (DST), where they created and recorded personal narratives using computers, adding images	To reduce stress in adolescent females through storytelling activities.	Self-report	Adolescent Stress Questionnaire (ASQ)

							and music. The intervention lasted six one-hour sessions over two months and was conducted in a high school setting. The focus was on emotional expression, peer support, and self-reflection, with some DST participants opting to keep their stories private.			
Hassen, 2022	Ethiopia	153	Control condition	Non-RCT (quasi-experimental design)	low	N/A	The mental health literacy program, a social media-based intervention. Using a quasi-experimental design, the study compared an intervention group, which received mental health education via Facebook and Telegram, to a control group that received unrelated educational content. Participants in the intervention group received summarized texts, figures, and case vignettes on mental health literacy, posted every 72 hours over six weeks. Topics covered mental health disorders, risk factors, self-help strategies, and help-seeking behaviours.	To improve adolescents' understanding of mental health issues.	Self-report	Mental Health Literacy Questionnaire
Hadley, 2019	United States	85	Comparative intervention group	RCT	high	School	The study tested an emotion regulation intervention delivered in two formats: Emotion Regulation + Immersive Virtual Reality Environments (ER + IVRE) - Participants practiced emotion regulation skills in realistic virtual scenarios (e.g., a party, condom purchasing, sexual negotiation, and HIV/STD testing). Emotion Regulation + Role-Play (ER + RP) - Participants practiced the same skills through traditional role-plays with facilitators and peers.	To improve emotion regulation (ER) skills in adolescents.	Self-report	Affect Dysregulation Scale

							Both interventions were group-based, delivered in four weekly 2-hour sessions, including didactic lessons, games, and skill-building exercises. The sessions focused on recognizing emotions, managing emotional triggers, and improving decision-making in risk situations.			
Huag, 2021	Switzerlan d	1473	Control condition	RCT	high	School	The SmartCoach program, a mobile phone-based life-skills intervention. It began with a brief classroom session for program introduction and app registration. Participants received 2-3 personalized text messages per week for 22 weeks, including quizzes, self-reflection tasks, and goal-setting exercises on stress management, self-control, and peer resistance. Automated personalized feedback guided skill application, with optional interactive challenges like self-monitoring stress and screen time.	To prevent substance use prevention in adolescents and foster life skills.	Self-report	Well-Being (WHO-5 Well-Being Index); Brief Interpersonal Competence Questionnaire (ICQ-10)
Kazemi, 2022	Iran	102	Control condition	RCT	high	School	A cognitive-behavioural educational program among female students aged 10-13 in Khomeini Shahr, Iran. Participants in the intervention group completed five weekly one-hour online sessions via the Shaad virtual learning platform. A trained instructor led the sessions, using storytelling and role-playing to help students recognize emotions, improve communication, and practice appropriate social behaviours. Each session focused on emotional awareness, managing impulsive behaviours, and practicing assertive communication. Students also	To improve social skills.	Self-report	Matson Evaluation of Social Skills with Youngsters (MESSY)

							completed home assignments, such as role-playing exercises, which they recorded and shared with their group.			
Ko, 2023	South Korea	60	Control condition	RCT	high	School	Koala DMT (Digital Mental Training) intervention is a brief, app-based program. It consists of ten sessions delivered over two weeks, with each session lasting approximately 7 minutes. The intervention focuses on lazy breathing exercises guided by animations through the Koala mobile app. Sessions were conducted in groups of 15 students during school lunch breaks, using tablet personal computers. The goal is to enhance mindfulness and emotional control in a school-based setting.	To improve emotional regulation and reduce stress in adolescents.	Self-report	State-trait anxiety inventory-X (STAI-X); Korean-mindful attention awareness scale (K-MAAS)
Lang, 2009	United States	55	Wait-list control	RCT	low	Community (computer labs within four affordable housing communities)	Computeen is a preventive computer and psychosocial skills development program designed for middle school students from a predominantly African American, low-income urban community. Participants attended structured group sessions where they engaged in computer-based learning activities, working individually on tasks related to word processing, internet use, and multimedia projects. Alongside the technical training, facilitators led psychosocial skill-building exercises, including group discussions and activities focused on decision-making, self-confidence, and social skills.	The improve technical computer skills and prevent maladaptive behaviours.	Both (Self-report – emotional; Caregiver-report – behavioural)	Rosenberg self-esteem scale; Child Behaviour Checklist

Lappalainen, 2021	Finland	243	Three groups: iACT+face-to-face support; iACT with online-only support; control group	RCT	high	School	The Youth COMPASS intervention was delivered in two formats: Virtual: Participants completed 10 weekly online modules (15–30 min each) on self-awareness, emotional regulation, and personal growth via a web-based platform. They received weekly asynchronous motivational feedback from coaches on WhatsApp, including reflective questions and encouragement. Hybrid: Participants completed the same 10 online modules but also had two in-person coaching sessions for personalized support. They received weekly WhatsApp feedback to reinforce engagement and self-reflection.	To prevent mental health problems in adolescents and promote adolescent well-being.	Self-report	Depression Scale (DEPS) and the Avoidance and Fusion Questionnaire for Youth (AFQ-Y) Depression Scale (DEPS) and the Avoidance and Fusion Questionnaire for Youth (AFQ-Y)
Lillevoll, 2014	Norway	707	Three groups: MoodGYM with tailored e-mail reminders; MoodGYM with standardized reminders; and MoodGYM with no reminders - or a waitlist control group	RCT	low	N/A	MoodGYM, a self-directed internet-based cognitive behavioural therapy (iCBT) program designed to prevent and reduce depressive symptoms in adolescents. Participants were assigned to one of three intervention groups: MoodGYM with tailored e-mail reminders (personalized based on depression risk, self-esteem, and self-efficacy). MoodGYM with standardized weekly e-mail reminders (general prompts about module completion). MoodGYM with no reminders (access to the program but no follow-up). There was also a waitlist control group. The intervention included five modules, each taking 30–45	To reduce depressive symptoms and promote self-esteem and self-efficacy.	Self-report	Center for Edpidemiologic Sudies Depression Scale (CES-D)

							minutes, covering topics like cognitive restructuring, emotional regulation, and behavioural activation.			
Liu, 2024	China	59	Wait-list control	RCT	low	N/A	The OKmind intervention is a 6-week, online mindfulness program for Chinese children aged 4-7 years, designed to improve attention and emotion regulation through a culturally adapted curriculum. Children attended six weekly 30-minute group sessions via the Tencent Meeting platform, led by trained facilitators. Sessions included mindfulness exercises, such as breathing techniques for focus and calmness, body scanning for physical awareness, and emotion regulation practices to manage anger and sadness. They also participated in creative expression activities, like drawing and storytelling, to reinforce mindfulness concepts.	To improve emotion and attention regulation.	Self-report	Anger dysregulated expression; Flanker reaction time
Manicavasagar, 2014	Australia	235	Control condition	RCT	low	N/A	The Bite Back program is a self-guided, web-based intervention grounded in positive psychology. Participants independently engage with: Interactive modules featuring exercises, reflection prompts, and quizzes. Educational content on positive psychology principles. Optional participation in online forums for peer discussions and resource sharing.	To improve mental well-being in adolescents and young adults by fostering skills such as gratitude, mindfulness, and optimism.	Self-report	Depression, Anxiety, and Stress Scale-Short-Form (DASS-21)
McRury, 2010	United States	51	Control condition	RCT	low	Community hospital	The calming intervention, a 30-minute instructional videotape, was designed to support parents of infants by demonstrating swaddling, side positioning,	To reduce infant crying.	Caregiver-report	Baby's Day Diary

							shushing, jiggling, and sucking techniques. Initially viewed in a hospital setting, the videotape was intended for independent, at-home use to help reduce infant crying and enhance parent-infant interactions. In addition to the videotape, parents received telephone check-ins to provide support, address questions, and reinforce the calming techniques.			
Mesurado, 2019	Argentina	51	Repeated-measures design	Non-RCT	low	University	The Hero Program, a self-administered online intervention, in adolescents aged 12-16. Participants completed seven online sessions (one per week) using a web-based platform, where they engaged with interactive exercises, videos, and gamified activities. The program featured a virtual guide ("Sensei"), who guided participants through tasks such as recognizing emotions in images, writing gratitude letters, practicing forgiveness through reflective exercises, and learning about helping behaviours.	To promote prosocial behaviour.	Self-report	Kindness and Generosity subscale from the Values in Action Inventory of Strengths
Mesurado, 2022	Argentina	756	Wait-list control	RCT	low	School	The Hero intervention is a school-based program for adolescents aged 12-15. Delivered in school computer rooms with health professionals present, the program included interactive online modules, scenario-based exercises, and self-reflection activities. Participants watched videos, engaged in decision-making tasks, and tracked their emotional growth, while health professionals provided guidance and support.	To promote prosocial behaviour.	Self-report	Prosocial behaviour towards strangers; Perspective taking

Morawska, 2006	Australia	110	Repeated-measured design	Non-RCT	low	N/A	A self-administered behavioural family intervention (BFI) designed for parents of toddlers, delivered through a telephone-assisted self-directed model within Parentline, a telephone counselling service. Parents received structured educational materials, including Every Parent's Self-Help Workbook, tip sheets on toddler behaviours, and the video Every Parent's Survival Guide. Over ten weeks, they completed workbook tasks and practiced parenting strategies, focusing on promoting social competence in children and managing difficult behaviours. Each week, parents participated in telephone consultations with trained counselors who encouraged self-regulation and independent problem-solving.	To promote children's social competence and strategies for dealing with difficult behaviour.	Caregiver-report	Eyberg Child Behavior Inventory
Nagamitsu, 2022	Japan	217	Three groups: Well-Care Visit (WCV) group; WCV + Smartphone CBT app group + control condition group	RCT	high	Hospital out-patient clinic	The study tested two adolescent health promotion interventions: A Well-Care Visit (WCV) and a smartphone-based cognitive behavioural therapy (CBT) app. The WCV group received in-person health checkups, structured counseling using the HEEADSSS framework, and educational materials on mental and physical health. The WCV + CBT app group received the same intervention plus access to a two-week CBT app, which provided psychoeducation and self-monitoring exercises to track thoughts, emotions, and behaviours. A nonintervention group received no immediate	To promote mental well-being and reduce depressive symptoms.	Self-report	Depression Self-Rating Scale for Children (DSRS-C); Adolescent Health Promotion Short Form (AHP-SF)

							treatment but was later offered the intervention.			
Neal-Barnett, 2019	United States	72	Repeated-measures design	Non-RCT	low	School	The Build Your Own Theme Song (BYOTS) app, designed to help Black and biracial adolescent girls manage anxiety and negative thinking through musical cognitive restructuring. 72 7th and 8th grade girls participated in Sisters United Now (SUN), an eight-session, culturally relevant anxiety intervention. As part of the program, participants were introduced to negative and positive thought cycles and learned how music could help interrupt negative thoughts. Each girl selected a favourite song and rewrote its lyrics using positive affirmations derived from a vision statement and a positive word bank. They then recorded their personalized theme song in the BYOTS app. They were reminded to use the app three times daily and encouraged to use it whenever they experienced negative thoughts.	To reduce negative thoughts.	Self-report	Multidimensional Anxiety Scale for Children 2 (MASC-2); pre post in-app survey on negative thought reduction validated by prior work
Nelson, 2002	United States	492	Repeated-measures design	Non-RCT	low	School	The Family Management Program is a comprehensive school-based program in seven elementary schools over two years. The program included school-wide behavioural support, one-to-one reading tutoring, conflict resolution training, and a video-based family management program. Students participated in school-wide behavioural interventions, one-to-one reading tutoring for those at risk, and conflict resolution training	To prevent problem behaviours and improve student learning.	Teacher-reported (social behaviour); School-recorded disciplinary and academic data	Behavioral and Emotional Rating Scale (BERS)

							through the "Talk It Out" program. Those with significant behavioural challenges received individualized intervention plans. Teachers led school-wide discipline and conflict resolution training, while graduate student tutors provided one-to-one reading support. Graduate students in social work assisted with conflict resolution, and a family intervention specialist guided parents through a video-based training program with phone consultations.			
Ohashi, 2024	Japan	44	Repeated-measures design (single-arm feasibility study)	Non-RCT	low	N/A	The Journey of the Brave intervention is an e-learning, CBT-based anxiety-prevention program for children aged 10-12. Delivered via a website, it includes eight stages covering emotional regulation, cognitive restructuring, and relaxation techniques. Participants watched videos, completed workbook exercises, and took quizzes. Gamification elements, such as earning points and unlocking characters, encouraged engagement. Parents monitored progress and completed surveys. The study assessed feasibility through participation rates, satisfaction, and changes in anxiety and emotional regulation skills.	To prevent anxiety in children and improve emotion regulation skills.	Self-report (SCAS); there is a SCAS-Parent version but did not use it.	Spence Children's Anxiety Scale Child Version (SCAS-S)
Pavarini, 2023	United Kingdom	100	Wait-list control	RCT	low	N/A	The Uplift Peer Support Training intervention is a virtual program designed to train individuals in peer support skills. The program consisted of 5 weekly live sessions conducted via Zoom, each lasting	To promote adolescents' emotional support skills, mental health and agency.	Self-report	Strengths and Difficulties Questionnaire - Emotional Symptoms Subscale (SDQ-E)

							approximately 2 hours. Participants learned and practiced peer support techniques through interactive modules, breakout room activities, and small group discussions. Independent tasks, including skill practice and reflection exercises, were assigned between sessions and supported through WhatsApp communication.			
Perry, 2017	Australia	540	Control condition (lifeSTYLE program)	RCT	high	School	The SPARX-R intervention is a cognitive behavioral therapy (CBT) program designed to prevent depression in final-year secondary school students. Delivered in a fantasy role-playing game format, the program guided participants through seven interactive modules, each lasting 20–30 minutes, where they learned and practiced CBT-based skills such as cognitive restructuring, emotion regulation, problem-solving, and behavioral activation. Additionally, students were given a paper notebook to record reflections and key takeaways from each module.	To reduce depressive symptoms.	Self-report	Major Depression Inventory; Depression Stigma Scale
Peuters, 2024	Belgium	279	Control condition; (2 intervention conditions – remote and in-school)	Non-RCT (two-arm cluster-controlled trial)	low	School	The #LIFEGOALS intervention is a mobile health program designed for adolescents aged 12-15. The program focuses on increasing physical activity, reducing sedentary time, improving sleep, and encouraging regular breakfast consumption. It is delivered through a self-guided mobile app that incorporates behaviour change techniques, gamification, and self-regulation tools. In the virtual format, participants engaged with	To improve mental well-being and promote healthy lifestyle behaviours.	Self-report	Moods & Emotions, and Self-Perception (KIDSCREEN-52); Psychological Well-being and Social Support & Peers (KIDSCREEN-27)

							the #LIFEGOALS app independently. The app provided self-regulation tools such as goal-setting, action planning, and self-monitoring using a Fitbit. Motivational reminders and weekly narrative health videos reinforced key health behaviours. A chatbot offered automated support by sending encouraging messages and guiding participants through challenges and goal-tracking. In the hybrid format, participants used the #LIFEGOALS app with additional in-person support at school. Researchers helped install the app and provided guidance on using the Fitbit for self-monitoring. Facilitators introduced the intervention and provided motivation through classroom banners with encouraging messages. However, participants retained autonomy in how they engaged with the app and were responsible for tracking their own progress.			
Pisani, 2024	United States	223	Control condition (information-only text messages)	RCT	low	School	The Text4Strength intervention is an automated text messaging program designed to help high school students manage their mental health and reinforce the Sources of Strength suicide prevention program. The intervention was delivered over nine weeks, with participants receiving one to two text messages per week focused on coping strategies, emotional regulation, and help-seeking behaviours.	To reduce suicidal behaviour and improve mental health skills.	Self-report	Emotional regulation; help-seeking from adults at school

							Participants: Read and engaged with personalized text messages based on their interests, such as favourite TV shows and music. Responded to interactive messages, which triggered follow-up texts reinforcing coping techniques and emotional support strategies. Accessed help resources by texting specific keywords, such as "helpinfo," when needed.			
Schoneveld, 2016	The Netherlands	136	Control video fam condition (Max and the Magic Marker)	RCT	high	School	The MindLight intervention is a neurofeedback-based video game. The program consisted of 5 one-hour sessions, where children played the game at school after school hours under supervision. The game uses immersive virtual environments, attention-shifting exercises, and relaxation techniques to teach children how to manage anxiety. Gameplay was fully independent, but sessions were supervised.	To reduce anxiety in children.	Self-report (SCAS); there is a SCAS-Parent version but did not use it.	Spence Children's Anxiety Scale (SCAS)
Sim, 2020	Australia	355 parents and 342 children	Control condition (educational factsheets)	RCT	high	N/A	The Parenting Resilient Kids (PaRK) program is a self-guided online intervention designed. It included up to 12 interactive modules, tailored to parents' baseline assessments, covering parenting techniques, behaviour management, resilience, and coping strategies. Parents completed modules at their own pace, engaging with educational content, interactive activities, quizzes, and goal-setting exercises. To support participation, research personnel conducted brief weekly check-in calls for technical assistance,	To help parents reduce anxiety and depression risk in their children.	Self-report (SCAS); there is a SCAS-Parent version but did not use it.	Revised Children's Anxiety and Depression Scale (RCADS-25); KIDSCREEN-27

							without providing therapeutic advice or individualized parenting guidance.			
Skeen, 2023	Tanzania and Zambia	494 (caregiver-child dyads)	Wait-list condition	RCT	low	N/A	The Sharing Stories parenting intervention is a six-week digital parenting program delivered remotely via WhatsApp groups to caregivers in Tanzania and Zambia. The intervention included two key components: Promoting shared reading to encourage parent-child interaction and improving caregiver mental health by addressing negative thought patterns and stress management. Caregivers participated in weekly facilitated group discussions where they received digital picture books and guidance on how to engage their children in interactive storytelling.	To promote child's emotional and social skills; improve responsive parenting behaviours; and caregiver mental health.	Caregiver-report	Strengths and Difficulties Questionnaire (SDQ); subscales for prosocial behavior, emotional symptoms, hyperactivity, conduct problems, and peer problems.
Sousa, 2020	Portugal	353	Control condition	Non-RCT (quasi-experimental)	low	School	The TeenPower intervention is a six-month program for adolescents aged 12-16 years. It combined a self-directed mobile health (mHealth) component with structured school-based psychoeducational sessions. Adolescents used the TeenPower mobile app to access interactive content on nutrition, physical activity, stress management, and healthy habits, allowing them to track behaviours, set goals, and receive personalized feedback.	To promote lifestyle behaviours in adolescents (e.g., stress management).	Self-report	Adolescent Lifestyle Profile (ALP) - Stress Management; and Interpersonal Relations
Subotic-Kerry, 2023	Australia	438	Repeated-measures design	Non-RCT (quasi-experimental)	low	N/A	Bite Back Mental Fitness Challenge is a web-based positive psychology program targeting adolescents aged 13-16. It aims to promote mental well-being by focusing on five key domains of positive psychology:	To improve help-seeking intentions and reduce anxiety and depressive symptoms.	Self-report	Generalized Anxiety Disorder Scale (GAD-2 and GAD-7); General Help-Seeking

							gratitude, mindfulness, connections, character strengths, and meaning and purpose. The program comprised 7 self-directed modules designed to be completed over six weeks. Each module included activities such as animations, reflective questionnaires, and exercises to foster resilience. The Bite Back website offered additional resources on topics like optimism, healthy lifestyles, and positive relationships, as well as links to external mental health support services.			Questionnaire (GHSQ)
Sun, 2022	China	390	Wait-list control	RCT	high	School	The smartphone app encouraged participants to engage in Sharing, Mind, and Enjoyment (SME) behaviours through daily and special tasks, such as expressing gratitude, spending time with family, and completing activities together. Participants earned digital tokens for completing tasks, which could be used to decorate a virtual “dream house” or play mini-games. An introductory workshop, led by a social worker, introduced students to the app and key mental health concepts.	To promote mental well-being and increase awareness of anxious symptoms among adolescents.	Self-report	Sharing, Mind, and Enjoyment (SMEa) Behaviours; Short Warwick-Edinburgh Mental Well-being Scale (SWEMWBS); Personal and Family Health and Happiness (validated in Hong Kong Chinese populations)
Waters, 2019	Australia	303	Two intervention conditions: PST and CBI; and a Control condition	RCT	high	School	The intervention compares two classroom-based programs for reducing anxiety in children aged 7-11 years: Positive Search Training (PST): A computer-assisted program that trained children to focus attention on positive stimuli while ignoring negative stimuli. It involved searching for “good” (e.g., happy children) or “calm” (e.g.,	Reducing anxiety.	Self-report – (SCAC-C); Parent-report (Tbias)	Spence Children’s Anxiety Scale (SCAS); Attention Bias (Visual Probe Task)

							serene objects) images in arrays of distracting negative images. PST consisted of 8 sessions, each lasting 30 minutes, conducted twice weekly over 4 weeks. Cognitive Behavioural Intervention (CBI): A therapist-led program based on cognitive-behavioural principles. It included psychoeducation about anxiety, relaxation training, positive self-talk, social skills, and graded exposure exercises. Like PST, it was delivered over 8 sessions lasting 30 minutes each, conducted twice weekly for 4 weeks. The PST sessions were self-directed on school laptops, while the CBI was facilitated by trained clinical psychologists and did not involve digital tools.			
Waters, 2023	Australia	66	Repeated-measures design	Non-RCT	low	N/A	The intervention is part of the RISE Rugby League Development Program. It includes three core steps: Participants completed online measures of mental health and well-being. Parents received individualized feedback via telephone, including support and referral options if needed. Group-based workshops were delivered via teleconference during rugby training sessions, focusing on topics such as healthy habits, mindfulness, grit, gratitude, and social connection. The intervention spanned the rugby season, with four 30-40 minute workshops and ongoing independent access to psychoeducational resources.	To promote adolescent mental health and well-being.	Self-report	Revised Children's Anxiety and Depression Scale (RCADS-25); Strengths and Difficulties Questionnaire (SDQ); Academic Grit Scale; Prosocial Behaviours Subscale

Waters, 2024	Australia	671	Repeated-measures design; Three intervention conditions: In-person + remote real-time delivery; In-person + remote pre-recorded delivery; fully remote delivery	Non-RCT (quasi-experimental)	low	Rugby league clubhouses	The Life-Fit Learning System within the RISE Rugby League Development Program was delivered across three groups, combining in-person and remote methods: (a) In-Person + Remote Real-Time: Participants attended four hybrid workshops (30–40 min each), alternating between in-person sessions led by local well-being officers and live remote video sessions conducted by Life-Fit psychologists. They also completed self-assessments and accessed psychoeducational Tip Sheets. Parents received mental health feedback via email or phone. (b) In-Person + Remote Prerecorded: In-person workshops were led by well-being officers, while remote content was delivered via pre-recorded videos by Life-Fit psychologists. Participants engaged in four mixed-format workshops, supplemented with assessments and psychoeducational resources. Parents received feedback and referrals remotely. (c) Remote Only: All workshops were delivered remotely, either live via Microsoft Teams or through pre-recorded videos. Participants completed four remote sessions, self-assessments, and accessed additional resources. Parents received remote feedback and referral support.	To promote adolescent mental health and well-being.	Self-report	Revised Children's Anxiety and Depression Scale (RCADS-25); Strengths and Difficulties Questionnaire (SDQ)
Whittaker, 2017	New Zealand	855	Control condition (placebo condition)	RCT	high	Schools	The MEMO CBT intervention is a mobile phone-based program delivered over nine weeks through	To prevent depression in adolescents.	Self-report	Reynold's Adolescent Depression Scale-

							daily multimedia messages. Participants received two messages per day outside of school hours. These messages contained links to videos or text-based content. They watched short videos (less than 30 seconds) featuring teen actors, cartoons, and celebrities, which demonstrated cognitive-behavioural therapy (CBT) strategies such as managing negative thoughts, problem-solving, and emotional regulation. Participants engaged independently with the content by clicking on links to view materials and reflect on the messages. After completing the program, they had continued access to a mobile website that summarized key messages and provided additional mental health resources.			Second Edition (RADS-2)
Wolchik, 2022	United States	240	Wait-list control	RCT	high	N/A	The New Beginnings Program (eNBP), a parenting intervention, was designed to support divorced and separated parents. Adapted from the in-person New Beginnings Program (NBP), it consisted of 10 self-paced modules completed over approximately five hours. Participants engaged with interactive exercises, instructional videos, and automated reminders to learn and apply parenting strategies. The program focused on strengthening parent-child relationships, improving discipline, and reducing children's exposure to parental conflict. Parents were expected to complete home practice assignments and received	To reduce interparental conflict, improve parenting quality, and improve children's post-divorce adjustment.	Both; but used only self-report	Brief Problem Monitor (BPM); Strengths and Difficulties Questionnaire (SDQ)

							automated email and text reminders to reinforce learning.			
Wong, 2014	Australia	976	Two intervention conditions: anxiety prevention program; depression prevention program; and usual health classes control condition	RCT	low	School	The ThisWayUp Schools Depression and Anxiety Prevention Program is an internet-delivered, school-based cognitive-behavioural therapy (CBT) intervention. It consisted of 6 anxiety-focused lessons and 7 depression-focused lessons, each lasting 40 minutes and delivered over 6-7 weeks. Students accessed the program via a secure online platform and completed self-directed lessons that incorporated interactive elements to demonstrate CBT techniques. The intervention was implemented in schools during regular health classes, with teachers supervising the process and reinforcing learning through class discussions and worksheets.	To prevent anxiety and depression in adolescents.	Self-report	Generalized Anxiety Disorder Scale (GAD-7); Patient Health Questionnaire - Short Form (PHQ-5)
Wong, 2020	China	67 parent-child pairs (134 participants in total)	Repeated-measures design	Non-RCT	low	N/A	The Family Move app-based intervention is an 8-week program designed to encourage physical activity and improve parent-child engagement through a mobile app. The program includes guided exercises for parents and children to perform together, along with features such as points, leaderboards, and rewards to promote consistent engagement. Regular prompts were provided to encourage app usage and physical activity.	To promote the health and well-being of both children and parents through parent-child exercises.	Caregiver-report	Strengths and Difficulties Questionnaire (SDQ); subscales for prosocial behaviour, emotional symptoms, hyperactivity, conduct problems, and peer problems.
Zagni, 2024	United States	813	Control condition	RCT	low	School	PeerLearning.net is a web-supported cooperative learning program. Delivered over 10 weekly lessons, each lasting 30 minutes. Facilitated by teachers using	To improve mental health and well-being among students.	Self-reported and teacher-reported (only used self-report)	Strengths and Difficulties Questionnaire (SDQ); Peer Relations Scale

							structured lesson plans, templates, and tools provided by the PeerLearning.net platform. Students participated in cooperative learning activities, engaging in group tasks and individual exercises to reinforce skills.			
Zhang, 2023	China	160	Control condition	RCT	low	N/A	A digital Guided Self-Help Mindfulness-Based Intervention (GSH-MBI) was delivered via the WeChat mini program to support pregnant women in reducing stress and improving mindfulness, with effects on infant well-being at 6-weeks and 6-months. The program consisted of 8 sessions, each lasting 10–15 minutes, with video lessons and audio-guided practices focused on mindfulness techniques, such as breathing exercises, body scans, and self-compassion. Participants accessed the program independently and at their convenience through the WeChat mini program. At 6 weeks postpartum, measures assessed the potential benefits for infants, including temperament and developmental behaviours.	To prevent maternal psychological distress and support infant neuropsychological development.	Caregiver-report	Ages and Stages Questionnaire (ASQ-3); Early Infancy Temperament Questionnaire (EITQ)
Zheng, 2021	China	954	Control condition	RCT	low	N/A	The REAP intervention is a peer-to-peer live-streaming digital program. Participants in the intervention group received health education on the importance of physical activity and the 20-20-20 eye relaxation rule. They used the REAP app to upload videos and photos of their physical activities and relaxation exercises. During scheduled recess breaks, they engaged in self-led	To promote physical activity and reduce anxiety and eye strain among grade 7 homeschooled children during COVID-19.	Self-report	Spence Children's Anxiety Scale Child Version (SCAS-S)

							exercises by following pre-recorded workout videos. They followed the government-recommended break schedule, which included four 15-minute recesses per day. To encourage engagement, participants also received SMS reminders prompting them to exercise and practice relaxation techniques.			
Zulkefly, 2024	Malaysia	24 mothers of adolescents	Control condition	RCT	high	N/A	The Digital-Assisted Parenting Intervention (DaPI) consisted of 8 weekly sessions, each taking 30-40 minutes to complete. Delivered through a study-specific website, the program provided videos, infographics, comic strips, and interactive exercises to help parents improve communication, emotional support, and parenting self-efficacy. Participants received WhatsApp and email reminders to reinforce engagement.	To promote positive parenting practices and adolescent mental health.	Caregiver-report	CAPES (Child Adjustment and Parent Efficacy Scale); Behavioural Problems; Emotional Maladjustment Subscale

Supplementary Table 2. Representative Search Strategy

Ovid MEDLINE(R) and In-Process, In-Data-Review & Other Non-Indexed Citations <1946 to July 03, 2024>

#	Searches	Results	Type
1	(Promot* or prevent* or benefit* or develop* or reduce or help* or effectiveness or project or universal or experiences or (skill* adj2 build*) or (teacher adj2 led) or (school adj2 based) or "school-based" or "after school").ti.	1780389	Advanced
2	(trials or trial or intervention or app evaluat* or application* or (app* adj2 mental health) or (app* adj2 restructuring) or program* or (mobile adj2 app)).ti.	1095505	Advanced
3	(mind or mindful* or psychosocial* or (mental adj2 (health or well-being or wellness or "well being")) or (emotional* adj2 (health or well-being or wellbeing or wellness or "well being")) or resilien* or (cognit* adj2 (music or musical or skill or skills or ability or abilities)) or (life adj2 skill*) or behaviour* or behavior* or stress* or suicide or antecedents).ti.	882996	Advanced
4	(infan* or child* or adolescen* or youth* or student* or girl or girls or boy or boys or teen*).ti.	1496824	Advanced
5	1 and 2 and 3 [****Base clinical set no age groups*****]	8746	Advanced
6	limit 5 to "all child (0 to 18 years)"	2802	Advanced
7	6 or (4 and 5) [****Final results version 1*****]	3448	Advanced

Embase <1974 to 2024 July 03>

#	Searches	Results	Type
1	(Promot* or prevent* or benefit* or develop* or reduce or help* or effectiveness or project or universal or experiences or (skill* adj2 build*) or (teacher adj2 led) or (school adj2 based) or "school-based" or "after school").ti.	2139219	Advanced
2	(trials or trial or intervention or app evaluat* or application* or (app* adj2 mental health) or (app* adj2 restructuring) or program* or (mobile adj2 app)).ti.	1382020	Advanced

3	(mind or mindful* or psychosocial* or (mental adj2 (health or well-being or wellness or "well being")) or (emotional* adj2 (health or well-being or wellbeing or wellness or "well being")) or resilien* or (cognit* adj2 (music or musical or skill or skills or ability or abilities)) or (life adj2 skill*) or behaviour* or behavior* or stress* or suicide or antecedents).ti.	999199	Advanced
4	(infan* or child* or adolescen* or youth* or student* or girl or girls or boy or boys or teen*).ti.	1726409	Advanced
5	1 and 2 and 3 [****Base clinical set no age groups*****]	10069	Advanced
6	limit 5 to (infant <to one year> or child <unspecified age> or preschool child <1 to 6 years> or school child <7 to 12 years> or adolescent <13 to 17 years>)	3068	Advanced
7	6 or (4 and 5) [****Final results version 1*****]	3621	Advanced

APA PsycInfo <1806 to June Week 4 2024>

#	Searches	Results	Type
1	(Promot* or prevent* or benefit* or develop* or reduce or help* or effectiveness or project or universal or experiences or (skill* adj2 build*) or (teacher adj2 led) or (school adj2 based) or "school-based" or "after school").ti.	458946	Advanced
2	(trials or trial or intervention or app evaluat* or application* or (app* adj2 mental health) or (app* adj2 restructuring) or program* or (mobile adj2 app)).ti.	233428	Advanced
3	(mind or mindful* or psychosocial* or (mental adj2 (health or well-being or wellness or "well being")) or (emotional* adj2 (health or well-being or wellbeing or wellness or "well being")) or resilien* or (cognit* adj2 (music or musical or skill or skills or ability or abilities)) or (life adj2 skill*) or behaviour* or behavior* or stress* or suicide or antecedents).ti.	580825	Advanced
4	(infan* or child* or adolescen* or youth* or student* or girl or girls or boy or boys or teen*).ti.	800582	Advanced
5	1 and 2 and 3 [****Base clinical set no age groups*****]	7565	Advanced
6	limit 5 to (100 childhood <birth to age 12 yrs> or 200 adolescence <age 13 to 17 yrs>)	2628	Advanced
7	6 or (4 and 5) [****Final results version 1*****]	3392	Advanced

Preferred Reporting Items for Systematic reviews and Meta-Analyses extension for Scoping Reviews (PRISMA-ScR) Checklist

SECTION	ITEM	PRISMA-ScR CHECKLIST ITEM	REPORTED ON PAGE #
**TITLE**			
Title	1	Identify the report as a scoping review.	Abstract
**ABSTRACT**			
Structured summary	2	Provide a structured summary that includes (as applicable): background, objectives, eligibility criteria, sources of evidence, charting methods, results, and conclusions that relate to the review questions and objectives.	Abstract
**INTRODUCTION**			
Rationale	3	Describe the rationale for the review in the context of what is already known. Explain why the review questions/objectives lend themselves to a scoping review approach.	Introduction
Objectives	4	Provide an explicit statement of the questions and objectives being addressed with reference to their key elements (e.g., population or participants, concepts, and context) or other relevant key elements used to conceptualize the review questions and/or objectives.	Introduction
**METHODS**			
Protocol and registration	5	Indicate whether a review protocol exists; state if and where it can be accessed (e.g., a Web address); and if available, provide registration information, including the registration number.	Methods
Eligibility criteria	6	Specify characteristics of the sources of evidence used as eligibility criteria (e.g., years considered, language, and publication status), and provide a rationale.	Introduction
Information sources*	7	Describe all information sources in the search (e.g., databases with dates of coverage and contact with authors to identify additional sources), as well as the date the most recent search was executed.	Introduction/Methods
Search	8	Present the full electronic search strategy for at least 1 database, including any limits used, such that it could be repeated.	Supp. Table 2
Selection of sources of evidence†	9	State the process for selecting sources of evidence (i.e., screening and eligibility) included in the scoping review.	Methods
Data charting process‡	10	Describe the methods of charting data from the included sources of evidence (e.g., calibrated forms or forms that have been tested by the team before their use, and whether data charting was done independently or in duplicate) and any processes for obtaining and confirming data from investigators.	Methods
Data items	11	List and define all variables for which data were sought and any assumptions and simplifications made.	Methods
Critical appraisal of individual sources of evidence§	12	If done, provide a rationale for conducting a critical appraisal of included sources of evidence; describe the methods used and how this information was used in any data synthesis (if appropriate).	Results
Synthesis of results	13	Describe the methods of handling and summarizing the data that were charted.	Methods

SECTION	ITEM	PRISMA-ScR CHECKLIST ITEM	REPORTED ON PAGE #
**RESULTS**			
Selection of sources of evidence	14	Give numbers of sources of evidence screened, assessed for eligibility, and included in the review, with reasons for exclusions at each stage, ideally using a flow diagram.	Methods
Characteristics of sources of evidence	15	For each source of evidence, present characteristics for which data were charted and provide the citations.	Tables 1-5
Critical appraisal within sources of evidence	16	If done, present data on critical appraisal of included sources of evidence (see item 12).	Methods
Results of individual sources of evidence	17	For each included source of evidence, present the relevant data that were charted that relate to the review questions and objectives.	Results
Synthesis of results	18	Summarize and/or present the charting results as they relate to the review questions and objectives.	Results
**DISCUSSION**			
Summary of evidence	19	Summarize the main results (including an overview of concepts, themes, and types of evidence available), link to the review questions and objectives, and consider the relevance to key groups.	Discussion
Limitations	20	Discuss the limitations of the scoping review process.	Discussion
Conclusions	21	Provide a general interpretation of the results with respect to the review questions and objectives, as well as potential implications and/or next steps.	Discussion
**FUNDING**			
Funding	22	Describe sources of funding for the included sources of evidence, as well as sources of funding for the scoping review. Describe the role of the funders of the scoping review.	Acknowledgme nts

JBI = Joanna Briggs Institute; PRISMA-ScR = Preferred Reporting Items for Systematic reviews and Meta-Analyses extension for Scoping Reviews.
* Where *sources of evidence* (see second footnote) are compiled from, such as bibliographic databases, social media platforms, and Web sites.
† A more inclusive/heterogeneous term used to account for the different types of evidence or data sources (e.g., quantitative and/or qualitative research, expert opinion, and policy documents) that may be eligible in a scoping review as opposed to only studies. This is not to be confused with *information sources* (see first footnote).
‡ The frameworks by Arksey and O'Malley (6) and Levac and colleagues (7) and the JBI guidance (4, 5) refer to the process of data extraction in a scoping review as data charting.
§ The process of systematically examining research evidence to assess its validity, results, and relevance before using it to inform a decision. This term is used for items 12 and 19 instead of "risk of bias" (which is more applicable to systematic reviews of interventions) to include and acknowledge the various sources of evidence that may be used in a scoping review (e.g., quantitative and/or qualitative research, expert opinion, and policy document).

From: Tricco AC, Lillie E, Zarin W, O'Brien KK, Colquhoun H, Levac D, et al. PRISMA Extension for Scoping Reviews (PRISMA-ScR): Checklist and Explanation. Ann Intern Med. ;169:467–473. doi: 10.7326/M18-0850
